# Enzyme‐Substrate Complex Formation and Electron Transfer in Nitrogenase‐Like Dark‐Operative Protochlorophyllide Oxidoreductase (DPOR)

**DOI:** 10.1002/open.202500153

**Published:** 2025-04-08

**Authors:** Giada Bedendi, Plinio Maroni, Ross D. Milton

**Affiliations:** ^1^ Department of Inorganic and Analytical Chemistry Faculty of Science University of Geneva Quai Ernest-Ansermet 30 1205 Geneva Switzerland

**Keywords:** DPOR, Mechanism, Enzyme, Biocatalysis, Kinetic

## Abstract

Nitrogenase‐like dark‐operative protochlorophyllide oxidoreductase (DPOR) is a two‐component metalloenzyme involved in (bacterio)chlorophyll biosynthesis. DPOR enables photosynthesis in photosynthetic bacteria by catalyzing the MgATP hydrolysis‐dependent, stereoselective two‐electron reduction of protochlorophyllide (Pchlide) to chlorophyllide (Chlide). This requires the repeated transient association of DPOR's two component proteins (BchL and BchNB), and involves a series of individual and unresolved sequence of events (including MgATP‐hydrolysis, electron transfer, protein association/dissociation, substrate binding, *etc*.). DPOR shares structural and mechanistic similarities with nitrogenase, although the spectroscopic properties of Pchlide and Chlide permit the reaction to be followed *in situ* with visible spectroscopy. Here, we investigate DPOR's mechanism through vis‐spectroscopy in the absence of an electron donor in the system, where we were able to observe the formation of the enzyme–substrate (ES) complex prior to substrate reduction (electron transfer and MgATP hydrolysis). The determination of rate constants for ES formation as well as overall electron transfer reveals the complex rate‐limiting interplay between these two processes. Further, we observe evidence of cooperativity for ES complex formation in DPOR, which may be the origin of cooperativity during enzymatic turnover.

## Graphical abstract

Dark‐operative protochlorophyllide oxidoreductase (DPOR) participates in photosynthesis by catalyzing the ATP‐dependent reduction of protochlorophyllide to chlorophyllide. Structurally and mechanistically resembling nitrogenase, DPOR′s mechanism was investigated using visible spectroscopy. We used the vis‐spectra of substrate, product and Enyzme – Substrate complex (prior to the electron transfer) to study the kinetics of the mechanism and we observed cooperativity in the process.

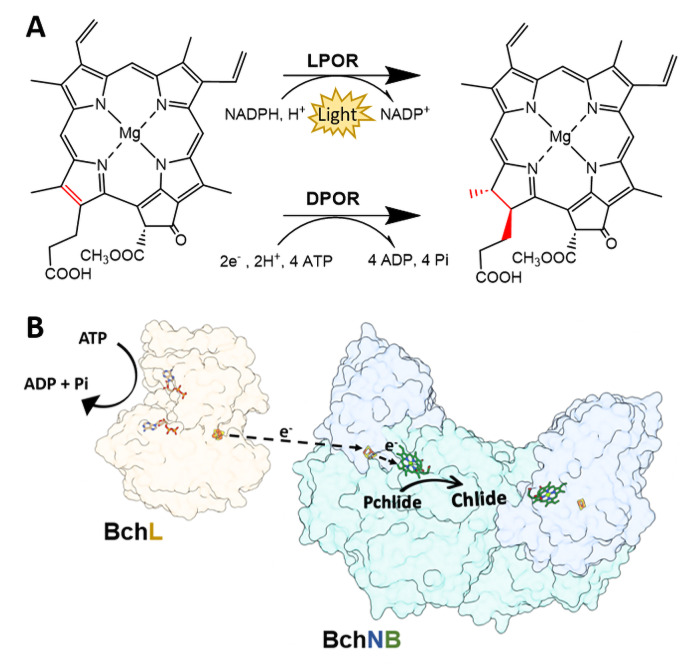

## Introduction

**Figure 1:**
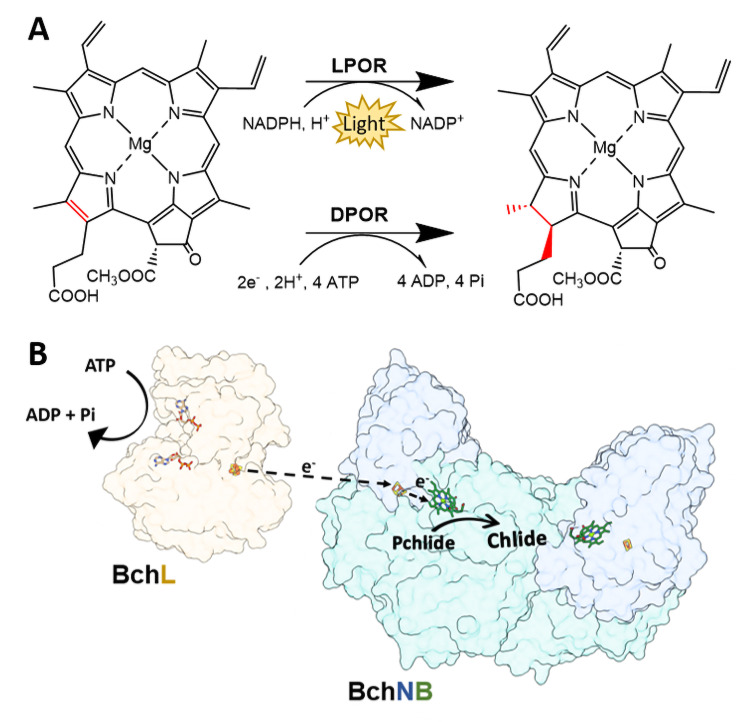
Reaction scheme for the reduction of protochlorophyllide (Pchlide) to chlorophyllide (Chlide) by DPOR. (B) Representation of DPOR′s X‐ray crystal structure (adapted from PDBs 3FWY (BchL from *Rhodobacter sphaeroides*) and 3AEK (BchNB from *R. capsulatus*)), where Fe=rust, S=yellow, N=blue, Mg=green, O=red.

The reduction of the D‐ring of protochlorophyllide (Pchlide) is a crucial step in the biosynthetic pathway of (bacterio)chlorophyll, where the double bond C_17_=C_18_ is reduced to yield chlorophyllide (Chlide, Figure 1A).[^1^, ^2^] Most photosynthetic organisms (*e. g*., cyanobacterium, algae and gymnosperm) are able to produce (bacterio)chlorophyll under both light and dark conditions. Pchlide reduction under light conditions is catalyzed by light‐dependent protochlorophyllide oxidoreductase (LPOR), an enzyme which depends on the nicotinamide adenine dinucleotide phosphate (NADPH) cofactor for electron delivery. In this case, photoexcitation in LPOR yields sufficiently reducing electrons for Pchlide reduction. Since Pchlide functions as a photoreceptor, the mechanism of LPOR relies on the excited‐state dynamics of Pchlide.[^3^, ^4^] In the absence of light, Pchlide reduction is catalyzed by dark‐operative protochlorophyllide oxidoreductase (DPOR, Figure 1B); in the place of photoexcitation in LPOR, DPOR couples the hydrolysis of adenosine triphosphate (MgATP) with electron transfer (ET) to catalyze Pchlide reduction. The overall mechanism of DPOR is not fully understood.^[5]^ Further, there are broad structural similarities between DPOR and its ancestral relative, nitrogenase. Surprisingly, while DPOR is found in both eukaryotic phototrophs and bacterial systems, nitrogenase is strictly limited to select bacteria and archaea.^[6]^

DPOR from the photosynthetic bacterium *Rhodobacter capsulatus* collectively consists of a 66 kDa MgATP‐hydrolyzing homodimeric γ_2_ reductase named BchL, and a 206 kDa Pchlide‐reducing heterotetrameric (αβ)_2_ protein named BchNB. Both of BchL's subunits contain a single MgATP‐binding site; two cysteine residues provided by each subunit coordinate a single subunit‐bridging [4Fe‐4S] cluster. Each αβ half of BchNB also contains a single [4Fe‐4S] cluster, coordinated by three cysteine residues from BchN and one aspartate residue from BchB (β‐D36 in *R. capsulatus*).[^7^, ^8^] Each αβ half also contains a single Pchlide‐binding site, comprising residues from both the α and β subunits, as well as the β’ subunit of the second αβ half. An aspartate residue on the opposing β’ subunit (β’‐D274) has been shown to be crucial to recognizing Pchlide and serving as a proton (H^+^) donor during Pchlide reduction.^[9]^

During turnover, BchL acts as a 1*e* ^−^ donor requiring the hydrolysis of 2MgATP. Thus, the 2*e* ^−^/2H^+^ stereospecific reduction of Pchlide on each αβ half of BchNB requires no less than two transient associations of the electron‐delivering BchL to the same half and the hydrolysis of 4MgATP. In DPOR each ET cycle includes the following events in an unresolved order: (i) the [4Fe‐4S] cluster of BchL is reduced by one electron (most likely by a ferredoxin or similar), (ii) 2MgATP binds to the BchL protein, (iii) Pchlide binds to BchNB (forming the enzyme‐substrate (ES) complex), (iv) reduced, MgATP‐bound BchL associates to the Pchlide‐bound BchNB protein, (v) a single electron is transferred from BchL's [4Fe‐4S] cluster through BchNB's [4Fe‐4S] cluster to Pchlide, (vi) hydrolysis of 2MgATP, (vii) dissociation of the BchL:BchNB protein complex, (viii) the release of inorganic phosphate (2P_i_) from BchL, and (ix) the release of 2MgADP from BchL. A second complete ET cycle results in the production and release of Chlide from BchNB.[^10^, ^11^, ^12^] Several studies of the related nitrogenase system have found that P_i_ release is the rate‐limiting step, although there is no such wide consensus in the case of DPOR.[^12^, ^13^, ^14^]

The association of MgATP to the analogous NifH protein of nitrogenase is known to lower the reduction potential (*E* ^0^’) of its [4Fe‐4S] cluster, thereby favoring ET to NifDK; while this is expected to be the case for DPOR, *E* ^0^’ values for DPOR's cofactors have not been reported to date.^[15]^ For both DPOR and nitrogenase, enzymatic activity assays, structural enzymology and computational studies have made clear that the two αβ halves engage in allostery with transient associations on one αβ half impacting the second one.[^16^, ^17^, ^18^, ^19^, ^20^] Unlike nitrogenases, substrate turnover (Pchlide reduction to Chlide) can be quantified directly *in situ* by visible spectroscopy.[^21^, ^22^]

In this paper, we used visible spectroscopy to follow the formation of the Pchlide:BchNB complex (enzyme‐substrate, ES‐complex), prior to initiating DPOR turnover by the addition of an electron donor. We observed that electron transfer from BchL to BchNB (or even a protein‐protein interaction) is not a prerequisite for Pchlide association to BchNB. Stepwise ES‐complex formation followed by Pchlide reduction allows us to measure the rate constants both steps, and reveals that the latter is the rate limiting step under commonly adopted *in vitro* turnover conditions. Additionally, we observed cooperativity behavior during ES‐complex formation, which suggests possible allosteric interactions prior to ET.

## Results and Discussion

Based on the distinct spectroscopic properties of Pchlide and Chlide, DPOR activity can be measured by visible spectroscopy as mentioned above. DPOR's standard activity assays are generally performed using 0.5–5 μM BchNB and at least a four‐equivalent molar excess of BchL reductase, in the presence of Pchlide (20–35 μM) and MgATP (~3 mM) in a Good's buffer (such as HEPES) at near‐neutral pH. Dithionite (DT, S_2_O_4_^2−^) is commonly used as the electron donor for DPOR (and is almost exclusively used for *in vitro* nitrogenase activation). Activity assays are usually quenched after about 8–10 minutes by the addition of acetone to a final 80 % v/v, the concentration of Chlide formed is calculated from absorbance at 666 nm using the extinction coefficient of 74.9 mM^−1^ cm^−1^.[^9^, ^23^, ^24^, ^25^, ^26^]

**Figure 2:**
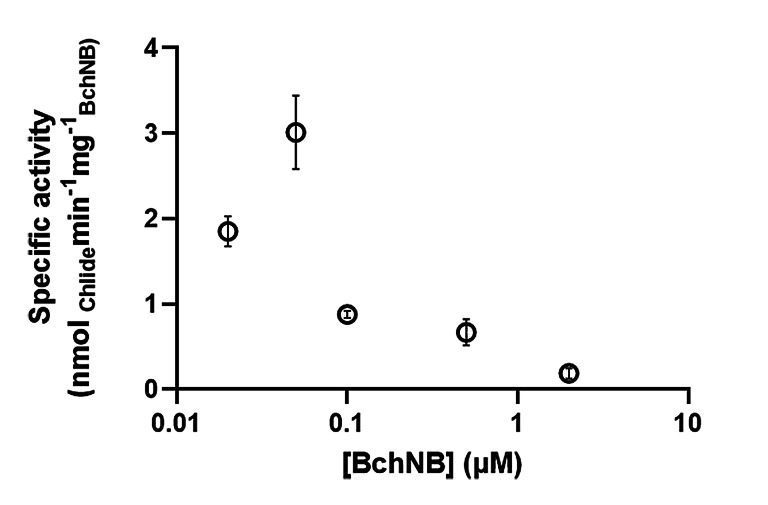
Specific activity assays were performed for 8 minutes and quenched in acetone (80% v/v final concentration). Each assay was conducted with varying concentrations of BchNB, a 4x molar excess of BchL, 20 μM Pchlide, and 2 mM DT. Data are presented as mean ± SD (*n*=3). Additional details are provided in the Experimental Section.

We first confirmed DPOR's activity by conducting several assays (details in the Experimental Section) maintaining the concentration of Pchlide fixed at 20 μM; we noted increased substrate precipitation at concentrations >20 μM Pchlide during long‐term *in situ* visible spectroscopic experiments. The specific activity was then evaluated over different concentrations of BchNB (2 μM, 0.5 μM, 0.1 μM, 0.05 μM and 0.02 μM, while always maintaining a 4 : 1 BchL : BchNB ratio). The results are reported in Figure 2 (further details can be found in Figure S1).

Importantly, we observed an increase in specific activities with the dilution of BchNB down to as low as 0.05 μM, which then diminished with further dilution. The highest activity recorded was 3.01±0.35 nmol_Chlide_ min^−1^ mg_BchNB_^−1^ (TOF=37.2 s^−1^) and we suggest that those conditions (0.05 μM BchNB) are ideal to saturate the enzyme. We note that it is challenging to perform traditional Michaelis‐Menten‐type analyses of DPOR, since we need a sufficiently large enough concentration of Pchlide to observe turnover, and concentrations above ~20 μM lead to substrate precipitation. We hypothesize that the decrease in DPOR specific activity at concentrations of BchNB <0.05 μM may be indicative of a substrate‐inhibition model, which could further complicate the development of such an *in vitro* kinetic model for DPOR. The coupling of DPOR with the next biosynthetic enzyme in *R. capsulatus* (Chlide‐reducing chlorophyllide oxidoreductase, COR) may be particularly important *in vivo*.

### In Situ Monitoring of Visible Absorbance

**Figure 3:**
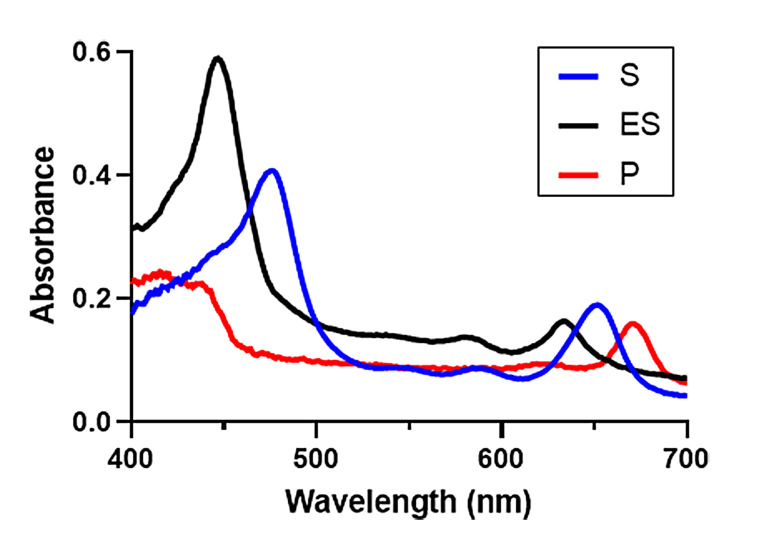
Reference spectra for S (blue, 8 μM), ES (black, 3.5 μM), and P (red, 5 μM), recorded in 100 mM HEPES buffer (pH 7.5) containing 150 mM NaCl, 3 mM ATP, 10 mM creatine phosphate, 3 mg creatine kinase and 10 mM MgCl_2_. Molar absorptivities (ϵ) used in data analysis are provided in Table 1.

Using a spectrophotometer within an anoxic chamber, we designed an experiment to monitor absorbance changes in real time in both the absence and presence of the DT electron donor. This permits us to determine kinetic parameters for the association of the Pchlide substrate to BchNB (ES complex formation) prior to Pchlide reduction. Figure 3 (and Figure S2) reports the visible absorption spectra of free Pchlide (substrate, S) and free Chlide (product, P), recorded in HEPES buffer used for DPOR's activity assays (detailed in the Experimental Section). As shown, the incubation of BchNB with Pchlide in the absence of DT and BchL leads to the formation of the ES complex.

The subsequent addition of the DT electron donor, the final component for enzymatic activity, induces the reduction of Pchlide to Chlide (ES to E + P). Previous studies on Pchlide bound to BchNB have been performed by Fujita *et al*., who isolated the ES complex by treating StrepTactin‐bound BchNB with Pchlide, before the ES complex was then eluted from the solid phase. Similar absorbance features were reported for the ES complex.^[27]^

As presented in Figures 3 and S2, S/ES/P have prominent absorbance features between 440–500 nm and 600–700 nm. In this work we focus principally on the features in the latter region, where transmittance in the experimental setup is greater and less susceptible to interference by precipitated components: Pchlide exhibits a prominent feature at 651 nm (ϵ_651_=23.4 mM^−1^ cm^−1^), which shifts to 633 nm upon ES complex formation (c_633_=31.1 mM^−1^ cm^−1^) and 670 nm upon the formation of Chlide/P (ϵ_670_=44.7 mM^−1^ cm^−1^) (these molar absorptivities are summarized in Table 1). We were unable to discern a visible spectrum for the formation of an EP complex, (only possible during turnover; the addition of isolated Chlide to free BchNB does not result in a new feature in the visible spectrum, which may reflect poor association of Chlide to BchNB and its quick release once formed (Figure S3)), thus, our enzymatic model disregards a sufficiently long‐lived EP intermediate step. We therefore assume a simple enzymatic model in which the ES complex is formed prior to electron delivery from BchL coupled with MgATP hydrolysis (Equation 1). Here, we assume a pre‐equilibrium between E + S and ES, where our data indicate that ES formation is highly favorable.

(1) E+S↔ES→E+P

**Table 1:** Molar absorptivities of substrate (S), enzyme‐substrate (ES) complex and product (P), in 100 mM HEPES buffer (pH 7.5) containing 150 mM NaCl, 3 mM ATP, 10 mM creatine phosphate, 3 mg creatine kinase and 10 mM MgCl_2_.

	Wavelength (nm)	ϵ (mM^−1^ cm^−1^)
**S**	651	23.4±1.1
**ES**	633	31.1±1.5
**P**	670	44.7±4.3

**Figure 4:**
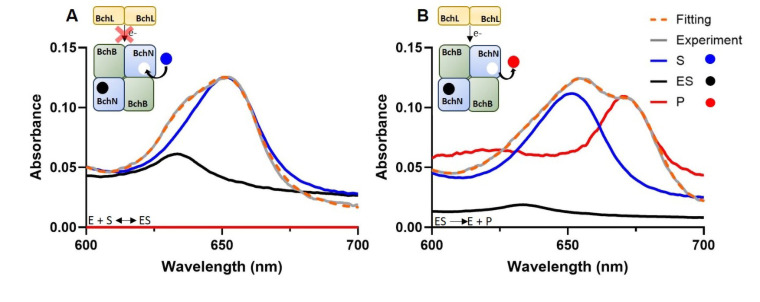
Parsing of the contributing components present in DPOR assays (E, ES and P) into their individual spectra by the use of an Octave script and their individual reference spectra as inputs (details in the Experimental Section). The orange dashed line represents the Octave fitting, the gray line shows the experimental absorbance data, the blue line indicates the substrate (S) contribution, the red line represents the product (P), and the black line corresponds to the enzyme‐substrate (ES) complex. (A) shows the fitting of ES complex formation, where the product contribution is zero. (B) displays the fitting of a spectrum where the product has formed, with a minimal ES complex contribution due to its conversion into the product. Representative experiment performed using 6.5 μM Pchlide and 0.5 μM BchNB, in 100 mM HEPES buffer (pH 7.5) containing 150 mM NaCl, 3 mM ATP, 10 mM creatine phosphate, 3 mg creatine kinase and 10 mM MgCl_2_.

The overlapping absorbance features of S/ES/P in aqueous solution complicates their individual analyses. To overcome this, we developed a script which is capable of extracting the S, ES and P contributions of the obtained absorbance spectra by using three reference spectra, corresponding to the absorption of isolated S, ES and P, at known concentrations (Figure S2) as inputs. Within the script, the reference spectra are spline‐smoothed and fitted (details in the Experimental Section). The visible spectra taken as reference for individual species (S, ES and P) were recorded in buffer. According to the reference spectra, the script corrects the proportionality coefficients of each reference spectra to obtain the best fitting for the recorded spectrum. Figure 4A and 4B demonstrates an example of the best fitting obtained for absorbance spectrum containing contributions of both the S + ES complex, or from a spectrum containing contributions from S, ES and P. The gray trace represents the original untreated absorbance data, while the orange dashed trace shows the best fit based on sum of the reference spectra. Using this method, we were able to measure the concentration of each species in every visible spectrum, following their formation or consumption over time. The method‘s success was later confirmed by verifying that the sum of the three concentrations remained constant throughout each DPOR activity measurement.

These real‐time experiments provide new insights into the catalytic mechanism, allowing us to determine rate constants for the formation of the ES complex (Pchlide‐bound BchNB) and its reduction to P (Chlide) *in situ*. As discussed above, these real‐time experiments were also restricted by the solubility of Pchlide but also the need to have sufficiently high concentrations of Pchlide and ES‐complex for their quantification. To combat this, the Pchlide concentration was limited to 20 μM although the concentration of BchNB was lowered to maintain its relative saturation by Pchlide under the conditions of our assays. A future study using an improved setup (i. e., a greater optical path length, etc.) may enhance the sensitivity of this experiment, along with its interpretation.

### In Situ Investigation of Kinetic Processes

**Figure 5:**
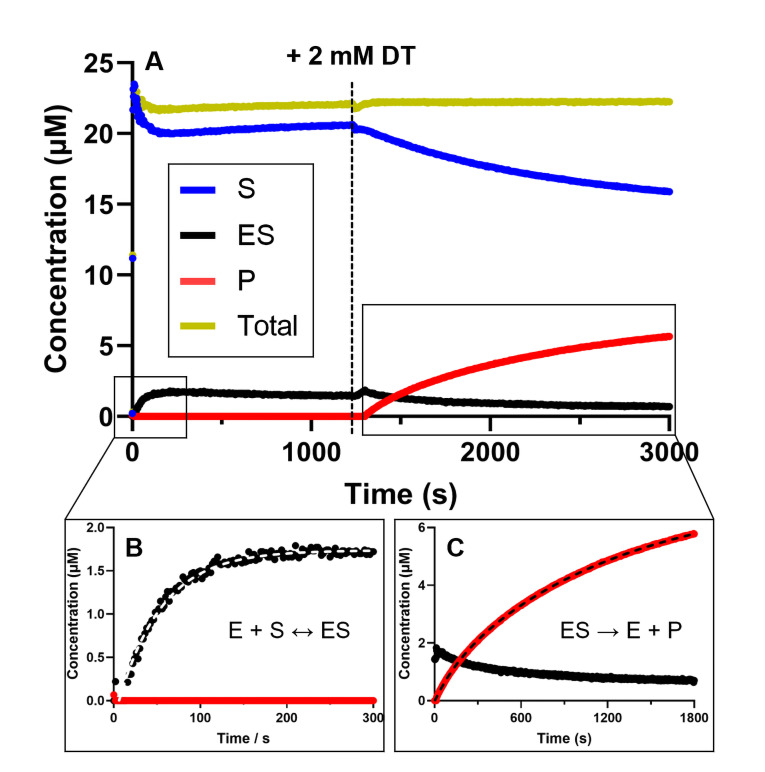
Representative time‐dependent concentration curve used to evaluate the rate constants for Enzyme‐Substrate (ES) complex formation and electron transfer (ET). These experiments were conducted in the presence of 0.5 μM BchNB, 2 μM BchL, and 20 μM Pchlide, with or without 2 mM DT. Absorbance was recorded over time and converted to concentration. A) The change in concentration over time is represented by the following traces: the substrate is shown in blue, the ES‐complex in black, the product in red, and the total concentration in yellow. B) the first phase of the experiment, during which the ES complex was allowed to form in the absence of the DT electron donor. Upon the addition of Pchlide, a decrease in free Pchlide concentration (blue trace, S) was observed alongside the formation of the ES complex (black trace, ES). C) illustrates the second phase, where DT was added to initiate ET for Pchlide reduction. This resulted in an increase in Chlide (P) formation over time, along with a corresponding decrease in the concentration of the ES complex.

To measure various rate constants during DPOR turnover, we initially designed an experiment in which absorbance was continuously recorded in the presence of 0.5 μM BchNB, 2 μM BchL, and 20 μM Pchlide, with or without 2 mM DT (Figure 5A). Under these conditions, the specific activity of DPOR was determined to be 850±218 pmol_Chlide_ min^−1^ (mg_BchNB_)^−1^ (as reported in Figure 2), consistent with values reported in the literature.[^28^, ^29^]

In the first phase of the experiment, we allowed the ES‐complex to form in the absence of the DT electron donor. This step corresponds to the initial part of equation 1 (Figure 5B):

### 1 E + S ↔ ES

The ES complex formation reaction was initiated by the addition of Pchlide. We observed an initial decrease in the concentration of free Pchlide (blue trace, S) alongside the formation of the ES complex (black trace, ES), as was expected. At this stage, without an electron donor, the reaction does not proceed to yield the product (red trace, P). The gold trace (labeled “Total”) serves to validate our data processing and confirms the accuracy of the molar absorptivities used here for E/ES/P. The concentration of the ES‐complex reached a peak of 1.64±0.20 μM within the first 300 seconds, before stabilizing at 1.3±0.16 μM. Since BchNB has two hypothesized binding sites (one per each αβ half), we expected the formation of 1 μM of the ES complex for 0.5 μM BchNB. In previous research on substrate‐binding studies on the related nitrogenase‐like enzymes DPOR and COR, a minimum of 1.1 (to a maximum of 1.4) moles of substrate interact with 1 mole of tetramer have been reported.^[30]^ We hypothesize that there may be unspecific binding sites on the BchNB tetramer although it remains unclear as to how this may impact DPOR's catalytic mechanism. The maximum rate constant for this step was determined to be *k*_ES_=(23±1)×10^−3^ s^−1^ and fits well to a single‐phase model.

After the incubation of BchNB with Pchlide and stabilization of the ES complex concentration, an excess of DT was introduced to initiate electron transfer between BchL and BchNB for the reduction of Pchlide (Figure 5C):

### 2 ES → E + P

**Figure 6:**
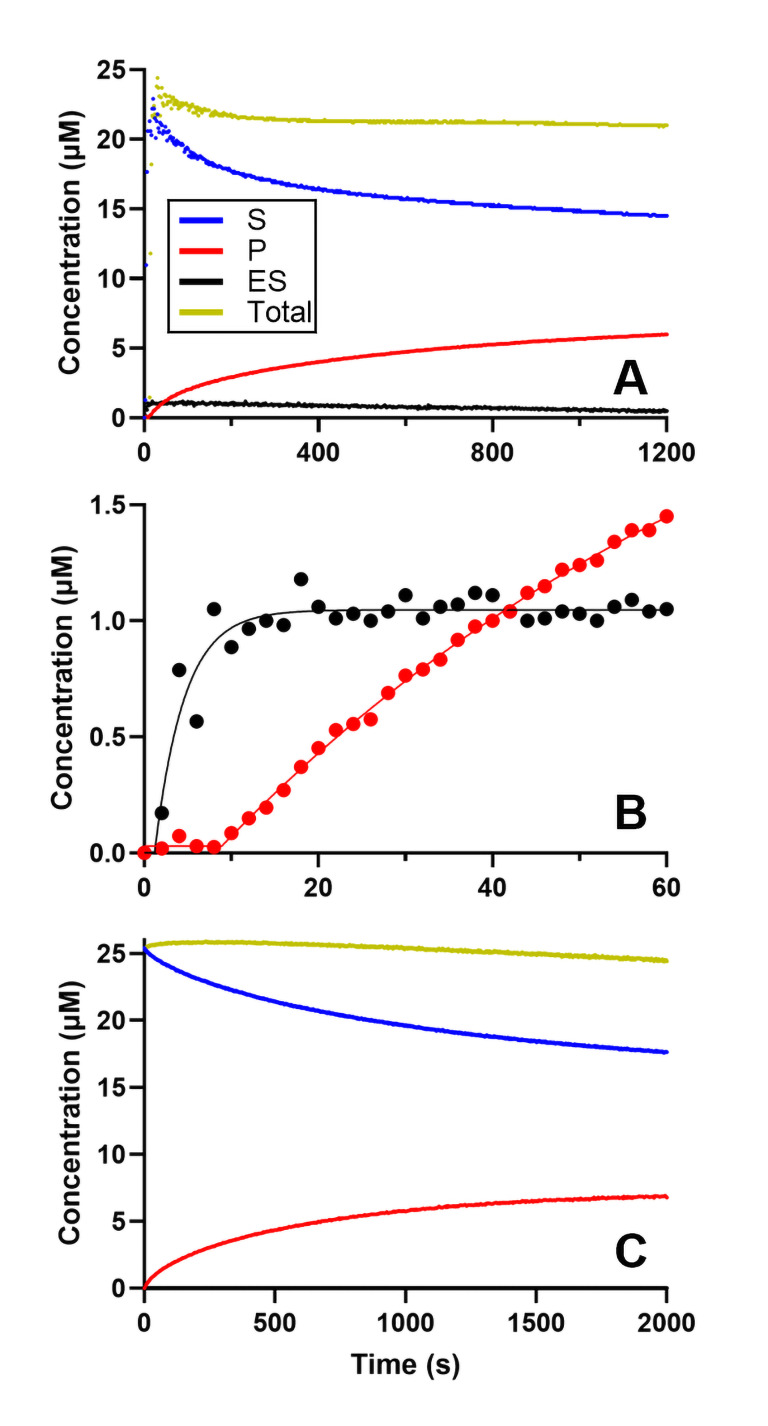
Representative time‐dependent concentration curves used to evaluate the kinetics constants during turnover conditions. These experiments were conducted with 0.5 μM BchNB, 2 μM BchL, and 20 μM Pchlide, along with 2 mM DT. Absorbance was recorded over time and converted to concentration. A) The reaction was initiated by the addition of Pchlide. The change in concentration over time is represented by the following traces: the substrate is shown in blue, the enzyme‐substrate (ES) complex in black, the product in red, and the total concentration in yellow. Panel B presents a window of the first minute of the reaction for the product and the ES‐complex formation. C) Data recorded by initiating the reaction with addition of BchNB.

We observed that P (Chlide) formation increases over time and is better described using a two‐phase fitting, yielding rate constants of *k*_P(fast)_=(4±1)×10^−3^ s^−1^ and *k*_P(slow)_=(0.6±0.2)×10^−3^ s^−1^. Interestingly, a decrease in the concentration of the ES‐complex was also observed. According to our data, the formation of Chlide is slower compared to the formation of ES complex under these conditions, and we therefore expect a steady state of the ES complex over the duration of this experiment. Subsequently, we investigated DPOR′s behavior under turnover conditions without the pre‐turnover formation of the ES complex. In this case, all necessary assay components were included and DPOR's reduction activity was initiated by the addition of Pchlide. As reported in Figures 6A and 6B, the ES complex was formed rapidly, reaching a concentration of 1.63±0.12 μM within 30 seconds, with a rate constant of *k*_ES_ ≥(230±20)×10^−3^ s^−1^; the instrument response time limits a more accurate determination of this rate constant (we could not measure ES‐complex formation in the first 10 seconds due to instrument limitations, as demonstrated in Figure S4). Interestingly, product (P, Chlide) formation was delayed (relative to ES‐complex formation) and was better described by a double‐phase kinetic model, with rate constants of *k*_P(fast)_=(12.2±2.6)×10^−3^ s^−1^and *k*_P(slow)_=(1.2±0.1)×10^−3^ s^−1^. Interestingly, the concentration of the ES complex continuously decreases towards 0 under these conditions. We hypothesize that this is due to the gradual deceleration of Chlide release as free Chlide begins to accumulate in solution (which would otherwise go on to be converted *in vivo* by COR). This was repeated by initiating turnover with the addition of BchNB (Figure 6C). In this case, we did not observe ES‐complex formation during DPOR turnover. Product formation once again followed a two‐phase kinetic model with *k*_P(fast)_ = (9±0.5)×10^−3^ s^−1^ and *k*_P(slow)_=(1±0.3)×10^−3^ s^−1^, consistent with the rate constants observed when the experiment was initiated by the addition of Pchlide.

Under these turnover conditions we observe that ES‐complex formation is not rate‐limiting. However, a comparison of the data summarized in Figures 5 and 6 indicates that *k*_ES_ increases by >10‐fold in the presence of the DT electron donor and MgATP. We previously excluded a MgATP hydrolysis‐dependent (and ET‐independent) mechanism, although the presence of both MgATP and DT alongside BchL and BchNB is expected to yield BchL:BchNB transient associations. We therefore propose two hypotheses for this observation: (i) BchL and BchNB undergo a preceding transient association whereby BchNB is pre‐reduced prior to ES‐complex formation, or (ii) *k*_ES_ is enhanced when BchL and BchNB are transiently associated (presumably prior to ET from BchL to BchNB).

#### Cooperativity Dynamics in DPOR

While it can be argued that multimeric proteins are normally assembled in nature to increase their functional stability, there are clear cases where multimer formation is employed to alter the function of proteins with respect to their monomeric counterparts. One celebrated example is that of myoglobin *vs*. hemoglobin; the assembly of α_2_β_2_ hemoglobin modifies the affinity of the heme oxygen‐binding centers across the protein as neighboring subunits bind molecular oxygen (O_2_). In the DPOR‐related enzyme nitrogenase, differing kinetic cooperativities have been reported in the literature.[^16^, ^17^, ^18^, ^19^, ^20^]

Early studies reported negative cooperativity, where electron transfer (ET) on one αβ half of nitrogenase's MoFe protein suppresses ET (and activity) in the other.^[16]^ Recent work has also reported positive cooperativity during steady‐state catalysis, particularly in the reduction of substrates like acetylene.^[17]^ In the case of DPOR, there is limited literature on cooperativity, although evidence of negative cooperativity during ET has been reported.^[12]^ We were able to adapt our experimental conditions to observe DPOR′s cooperativity behavior before ET occurs between BchL and BchNB.

**Figure 7:**
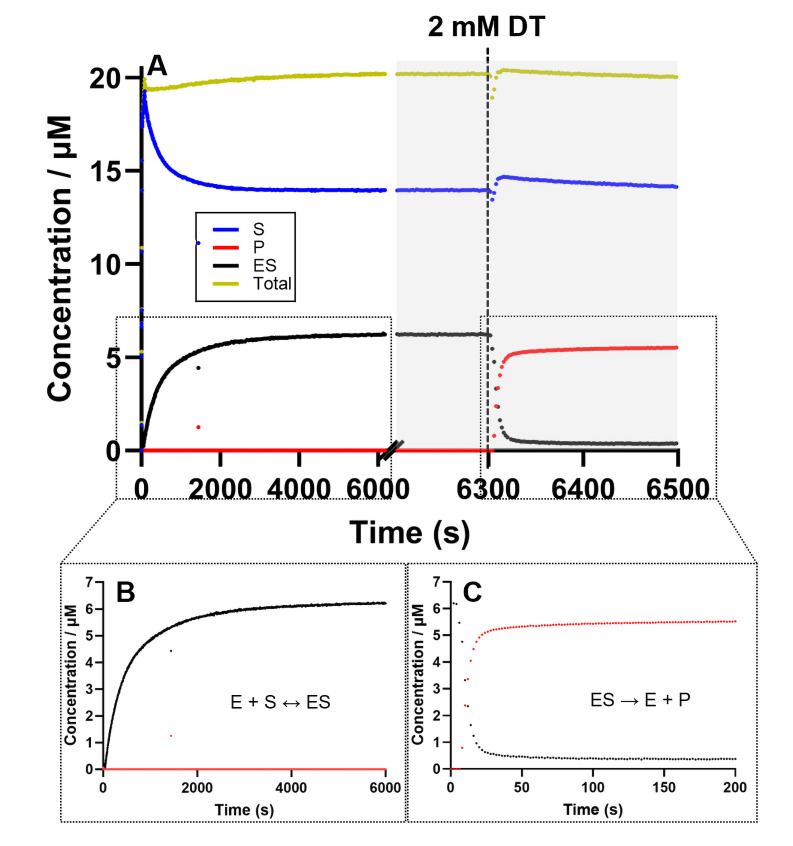
Representative time‐dependent concentration curve to investigate the behavior of DPOR during Enzyme‐Substrate (ES) complex formation and electron transfer (ET) when the system is limited by the concentration of Pchlide. These experiments were conducted in the presence of 2 μM BchNB, 8 μM BchL, and 20 μM Pchlide, with or without 2 mM DT. Absorbance was recorded over time and converted to concentration. The change in concentration over time is represented by the following traces: the substrate is shown in blue, the enzyme‐substrate (ES) complex in black, the product in red, and the total concentration in yellow. In the first phase, the ES complex was allowed to form in the absence of the DT electron donor. Upon the addition of Pchlide, a decrease in free Pchlide concentration (blue trace, S) was observed alongside the formation of the ES complex (black trace, ES). In the second phase DT was added to initiate Pchlide reduction. This resulted in an increase in Chlide (P) formation over time, along with a corresponding decrease in the concentration of the ES complex.

We conducted the real‐time experiments outlined above in the presence of a greater concentration of DPOR, using 2 μM BchNB, 8 μM BchL, 20 μM Pchlide, and 2 mM DT. The specific activity measured under these conditions by acetone quenching was 180±60 pmol min^−1^ mg_BchNB_^−1^ (Figure 2). As shown in Figure 7, the behavior differed from previous conditions with the ES‐complex now forming in two phases: *k*_fast_=(18±4)×10^−3^  s^−1^ and a slower, new *k*_slow_=(2.2±0.4)×10^−3^ s^−1^. The ES complex stabilized at 6.2±0.20 μM with there only being ~4 μM of expected Pchlide‐binding sites available. We hypothesize that this may be due to asymmetric Pchlide confirmations when BchNB is fully occupied,^[12]^ or perhaps unspecific (allosteric) additional association of Pchlide to BchNB. Next, DT was added to the solution to activate ET from BchL to BchNB and Pchlide reduction (ES turnover to P). *Note: DT is commonly used as an electron donor for DPOR and similar enzymes, but there is growing concern over non‐innocent or destructive roles that DT may play. Our recent work confirms that alternative electron donors to DT do not yield improved specific activity, and we therefore consider DT to be innocent here*.^[25]^

The concentration of the ES complex decreased and Chlide formation occurred immediately, with a rate constant of *k*_P_=(132.2±24.5)×10^−3^ s^−1^. Moreover, a Chlide plateau was reached while unreacted Pchlide was still present in the solution, with a concentration of 5.2±0.8 μM of Chlide obtained after 2.7±0.4 turnovers. The behavior is significantly different from our data above for the value of *k*_P_ and the number of possible turnovers.

**Figure 8:**
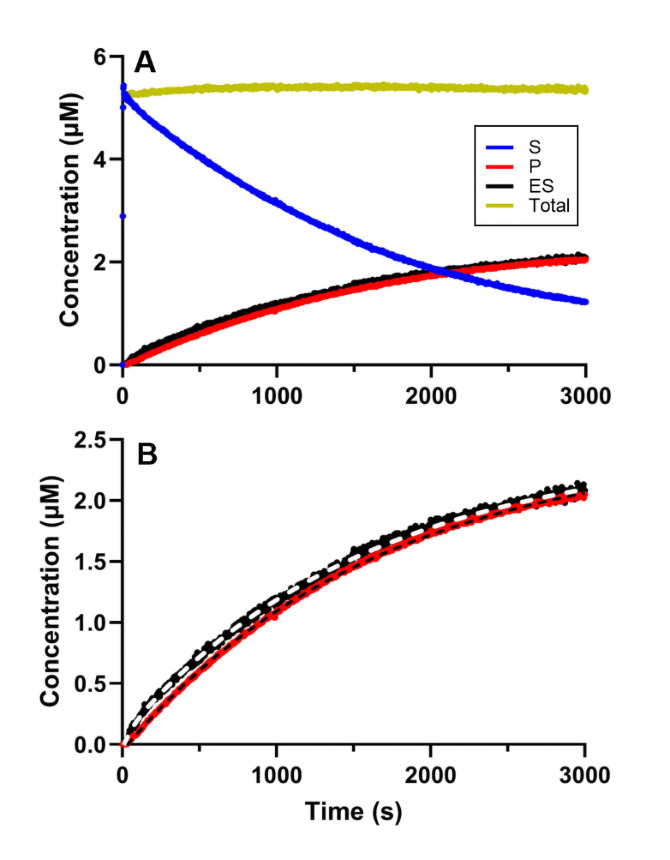
Representative time‐dependent concentration recorded under turnover conditions in the presence of a low concentration of Pchlide. This experiment was conducted with 2 μM BchNB, 8 μM BchL, and 6 μM Pchlide, along with 2 mM DT. Absorbance was recorded over time and converted to concentration. A) The reaction was initiated by addition of Pchlide. The change in concentration over time is represented by the following traces: the substrate is shown in blue, the enzyme‐substrate (ES) complex in black, the product in red, and the total concentration in yellow. In B) the zoomed view of the formation for the product and the ES‐complex.

These observations raised several questions: why do we have such a different behavior after changing the Pchlide:BchNB ratio? Why does the reaction cease after only a few turnovers? We do not have a clear explanation, but we can deduce that the ratio Pchlide:BchNB is crucial for future *in vitro* investigations. A relatively simple hypothesis is as follows. First, the concentration of BchNB is relatively increased, which further increases the probability of productively binding Pchlide (forming the ES complex) and leads to a faster formation of Chlide. However, given the reproducibility of the data, it seems unlikely that this behavior is explained by this hypothesis alone. We suggest that the different behavior is rather due to a different response of DPOR's regulatory mechanisms, in which the presence of Pchlide displaces Chlide. In these conditions we are limited by the concentration of Pchlide and most likely this limitation does not induce the release of Chlide, stopping the reaction after few turnovers. Moreover, prior to ET, the limiting Pchlide concentration concomitantly retards the formation of the ES complex. This was confirmed with a control experiment where 6 μM of Pchlide was used to initiate the reaction (all other necessary DPOR activity components were present). Figure 8 illustrates that the formation of both the ES complex and Chlide product proceeded at the same rate: 1.6±0.1 and 1.7±0.3 nmol s^−1^, respectively.

**Figure 9:**
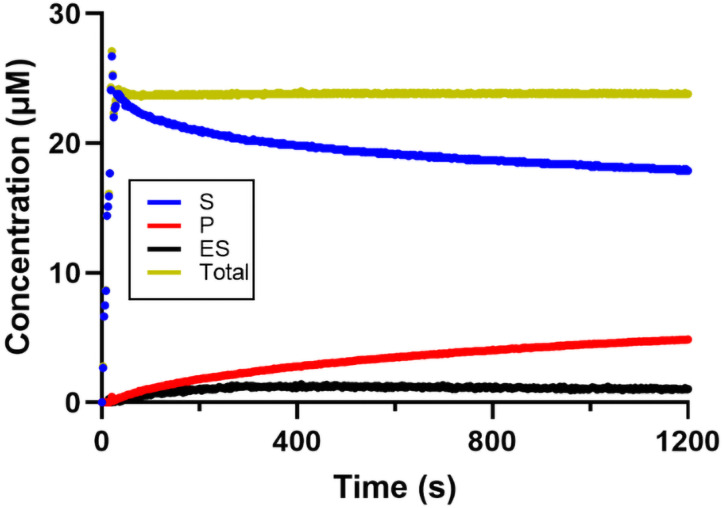
Representative time‐dependent concentration curve to evaluate DPOR behavior during turnover conditions when the system is limited by the concentration of Pchlide. These experiments were conducted with 2 μM BchNB, 8 μM BchL, and 20 μM Pchlide, in the presence of 2 mM DT. Absorbance was recorded over time and converted to concentration. The reaction was initiated by addition of Pchlide. The substrate is shown in blue, the enzyme‐substrate (ES) complex in black, the product in red, and the total concentration in gold.

Using the same conditions (limiting Pchlide concentration), DPOR's behavior was investigated under turnover conditions. The kinetic behavior we obtained was very similar to that observed for the formation of the ES complex under the same conditions. The reaction was initiated with Pchlide, resulting in a biphasic kinetic profile with *k*_fast_=(10.8±2.6)×10^−3^ s^−1^ and *k*_slow_=(1.4±0.2)×10^−3^ s^−1^ (Figure 9). We also observed the formation of the ES complex, supporting our hypothesis that turnover is stalled when low concentrations of Pchlide are present.

Under these conditions we were able to make ES‐complex formation the rate‐determining step of DPOR's mechanism and investigate it further. We observed a double‐phase kinetic response for ES complex formation, which was reproducible during turnover conditions. This double‐phase kinetic response supports the presence of communication between halves, which proceeds prior to ET and whose behavior may vary depending on the conditions used *in vitro*. Nevertheless, we are not able to clarify whether cooperativity is negative or positive at this stage.

## Conclusions

In this study, we explored the interaction of DPOR with its substrate, Pchlide, in the absence and presence of ET to BchNB, gaining insights into its mechanism using visible spectroscopy. Initially, we evaluated DPOR's activity, conducting the reaction under optimal buffer conditions while varying enzyme concentrations. Following standard activity assays reported in the literature, we measured maximal activity of DPOR using 0.05 μM BchNB and 20 μM Pchlide, yielding a specific activity of 3.01±0.35 nmol_Chlide_ min^−1^ mg_BchNB_^−1^ (TOF=37.2 s^−1^). Subsequently, we conducted *in situ* visible spectroscopy experiments to monitor absorbance changes in real‐time, which allowed for new mechanistic insights into DPOR. When BchNB is saturated with Pchlide, we observed rapid ES‐complex formation with a rate constant limited by the instrument‘s response time. Once the ES‐complex formed, an electron source was introduced to initiate ET whose kinetics exhibited slower biphasic behavior, making ET the rate‐limiting step. We conducted a comparative analysis of these values with those obtained under turnover conditions, specifically in scenarios where no pre‐formed ES complex was present. We observed that the kinetic profile was best described by a biphasic model, with rate constants of the same order of magnitude as in experiments where an electron donor (DT) was added after having completely formed the ES complex.

To better investigate the kinetics of ES complex formation, we conducted a second experiment with a lower Pchlide: BchNB ratio, where the limited Pchlide concentration slowed down the formation of the ES‐complex. We observed biphasic kinetics during ES‐complex formation. This finding suggests cooperativity begins during substrate binding, though further investigation is needed to determine if it is positive or negative.

Together, these findings indicate that DPOR activity and kinetics are highly sensitive to substrate availability, with ET emerging as a key rate‐determining step under saturation conditions. While we are able to identify cases where either ES complex formation or the overall ET cycle is rate‐limiting, we are currently unable to determine which is the rate‐limiting step under physiological conditions.

## Experimental Section

### BchL and BchNB Protein Purification

BchL and BchNB from *Rhodobacter capsulatus* were heterologously expressed in *E. coli*. Plasmids used for BchL and BchNB production were pHAL1 and pHANB1, with Strep tags inserted between codons 1 and 2. They were produced individually in *E. coli* BL21 *ΔiscR*, as previously reported.[^25^, ^26^] Purification was performed within an anoxic glovebox (COY Laboratory Products (Michigan, USA), [N_2_] >95 %, [H_2_] <5%, [O_2_] <5 PPM) using StrepTrap XT columns (5 mL, Cytiva, elution with 50 mM biotin) coupled to an Akta Go system.

### Extraction of Pchlide from Rhodobacter Capsulatus

Protochlorophyllide (Pchlide) was extracted from the culture super natant of *R. capsulatus* strain ZY5 (Δ*bchL*) and quantified in 80 % v/v acetone (ϵ_626nm_ = 30’400 M^−1^ cm^−1^), following the published protocol.^[31]^

### Activity Assays

Activity assays were performed in 100 mM HEPES buffer (pH 7.5) containing 150 mM NaCl, 3 mM ATP, 10 mM creatine phosphate, 3 mg creatine kinase, 10 mM MgCl_2_. Activity assays were performed within an Ar‐filled anoxic glovebox (Jacomex, France, with [O_2_] <1 ppm). Assays containing BchNB:BchL=1 : 4, 20 μM Pchlide and 2 mM sodium dithionite were performed in 100 μL final volume. Reactions were initiated by addition of BchL and quenched after 8 minutes of turnover at 22±1 °C by the addition of 400 μL acetone (80 % v/v final concentration). The reactions were then transferred to 1.5 mL Eppendorf tubes and centrifuged at 13,000×g for 10 minutes to remove the precipitated proteins. Finally, the supernatants were analyzed by UV/visible spectroscopy for the presence of Chlide (ϵ_666nm_=74’900 M^−1^ cm^−1^). Activity assays were performed using fixed concentrations/ratios of Pchlide and BchNB:BchL (1 : 4) ratio. All activity assays (*n*=3) contained a control in the absence of BchNB. Activity was subsequently calculated by individually subtracting the absorbance recorded in the control from each of the three repeats. For greater accuracy, GNU Octave was utilized to subtract the control absorbance. Excel was later used to evaluate the average and error of activity between the three repeats.

### In Situ Spectroscopic Measurement

Assays were run in 2 mL of 100 mM HEPES buffer (pH 7.5) containing 150 mM NaCl, 3 mM ATP, 10 mM creatine phosphate, 3 mg creatine kinase, and 10 mM MgCl_2_, at T=22 °C. Experiments were performed using 0.5 μM BchNB, 2 μM BchL, 20 μM Pchlide and ±2 mM sodium dithionite. Assays were performed in an Ar‐filled anoxic glovebox (Jacomex, France, [O_2_] <1 ppm) using and Autoloab UV/VIS/NIR spectrophotometer (Metrohm, Switzerland), fed‐through the glovebox using 200 μm fiber optic cables. Absorbance was recorded continuously. Turnover experiments were initiated either with Pchlide or BchNB. Cooperativity was investigated in the same environment with different concentration of enyzmes : 2 μM BchNB and 8 μM BchL.

### Reference Spectra

The absorption spectra of Pchlide (S), the ES complex (ES), and Chlide (P) were recorded in a Good's buffer (100 mM HEPES buffer (pH 7.5) containing 150 mM NaCl, 3 mM ATP, 10 mM creatine phosphate, 3 mg creatine kinase, 10 mM MgCl_2_), in the presence of BchNB for ES and P. All measurements were taken using a UV‐vis spectrophotometer with a 1 cm path length. The extinction coefficients of Pchlide and Chlide are reported in the literature for 80 % v/v acetone.^[27]^ For the reference spectra of ES, the extinction coefficient was initially calculated and later adjusted by ensuring that the total amount of Pchlide (free‐Pchlide + bound‐Pchlide) in solution was consistent over the experiment. The concentrations used for the determination of the reference spectra were 8 μM Pchlide, 3.5 μM for the ES complex and 5 μM for Chlide.

### Data Treatment

Data for rate constant determination and the investigation of cooperativity were treated using GNU Octave which can extract the *S*, *ES* and *P* contributions on the analyzed absorbance spectra by using as inputs three reference spectra corresponding to the absorption of isolated *S*, *ES* and *P* at known concentrations (Figure S2). Within the script, the reference spectra (not the data, only the reference spectra) are spline‐smoothed, and the following fitting function is created:

$$f\left(\lambda \right)=p_{1}\cdot S_{S}\left(\lambda -p_{6}\right)+p_{2}\cdot S_{P}\left(\lambda -p_{7}\right)+p_{3}\cdot S_{ES}\left(\lambda -p_{8}\right)+p_{4}+\frac{p_{5}}{\lambda^{3}}$$

Where *S*_S_, *S*_P_ and *S*_ES_ are the spline‐smoothed reference spectra for the substrate, product and complex respectively. The fitting function contains 7 fitting parameters. Parameters *p1, p2* and *p3* are the proportionality factors used to establish the contributions of each species in the analyzed spectrum, *p4* and *p5* take into consideration eventual baseline shift and possible precipitation in the sample. The remaining parameters are used to reflect potential wavelength shifts due to instability in the spectrophotometer. Each parameter can be set to run for optimization or to be fixed to a value. In our analysis, *p6, p7* and *p8* were fixed at 0 and noticeable wavelength shifts were not observed. The Octave function “non lin_curvefit” is used to best fit the function *f (λ*) to the analyzed spectrum and the result of the optimization is outputted at the end of the script. This curve was later fitted with GraphPad Prism by using exponential models including a plateau. For one phase kinetics the model used is “Plateau followed by one‐phase association” and for two‐phase kinetics, the model used is “two phase association”.

## Conflict of Interests

The authors declare no conflict of interest.

## Supporting information

As a service to our authors and readers, this journal provides supporting information supplied by the authors. Such materials are peer reviewed and may be re‐organized for online delivery, but are not copy‐edited or typeset. Technical support issues arising from supporting information (other than missing files) should be addressed to the authors.

Supporting Information

## Data Availability

The data and script(s) that support the findings of this study are openly available in Zenodo at https://doi.org/10.5281/zenodo.14284205, reference number 14284205.
