# Supplementary material for: Enzyme‐Substrate Complex Formation and Electron Transfer in Nitrogenase‐Like Dark‐Operative Protochlorophyllide Oxidoreductase (DPOR)
Source: ChemistryOpen. 2025 Apr 8;14(10):e202500153. doi: 10.1002/open.202500153 (PMC12518044; doi:10.1002/open.202500153)
Supplement: Supplementary file 1 — Supporting Information [file OPEN-14-e202500153-s001.pdf]

ChemistryOpen

Supporting Information

Enzyme-Substrate Complex Formation and Electron Transfer in Nitrogenase-Like Dark-Operative Protochlorophyllide Oxidoreductase (DPOR)

Giada Bedendi, Plinio Maroni, and Ross D. Milton*

Supplementary Information for:

Enzyme-substrate complex formation and electron transfer in nitrogenase-like dark-operative protochlorophyllide oxidoreductase (DPOR)

Giada Bedendi, Plinio Maroni, and Ross D. Milton*

Department of Inorganic and Analytical Chemistry, University of Geneva, Faculty of Sciences, Quai Ernest-Ansermet 30, 1205 Geneva 4, Switzerland

Corresponding Author

Email: ross.milton@unige.ch

Raw Data

All raw data are available on Zenodo (DOI: 10.5281/zenodo.14284205).

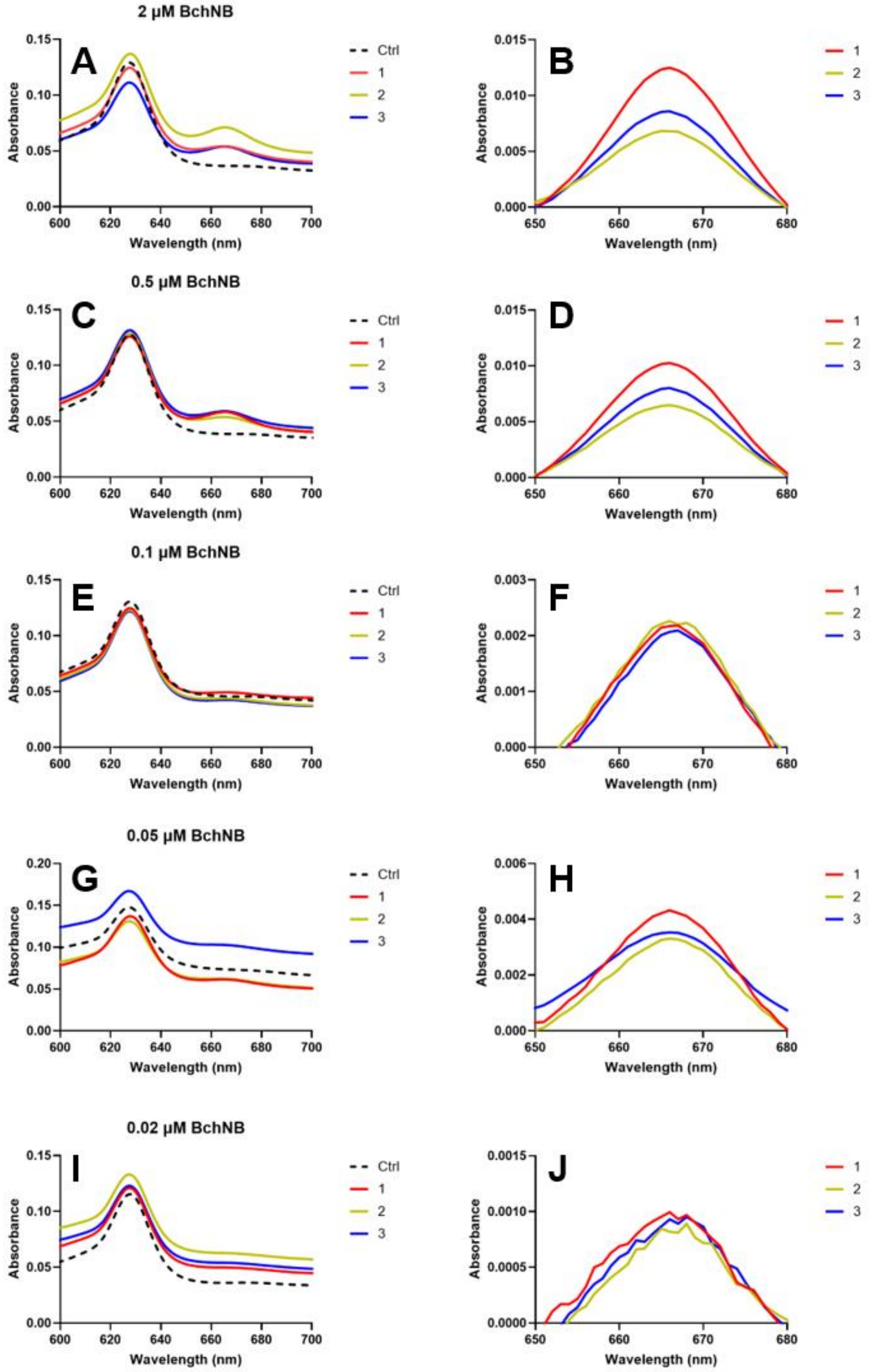

Figure S1. Specific activity assays were conducted for 8 minutes and then quenched in acetone. Each assay included X µM BchNB, 4X µM BchL, 20 µM Pchlide, and 2 mM DT. Figures A, C, E, G, and I display raw data, while Figures B, D, F, H, and J show processed data using Octave software. Assay conditions for each figures pair are as follows: (A-B) X = 2 µM, (C-D) X = 0.5 µM, (E-F) X = 0.1 µM, (G-H) X = 0.05 µM, and (I-J) X = 0.02 µM.

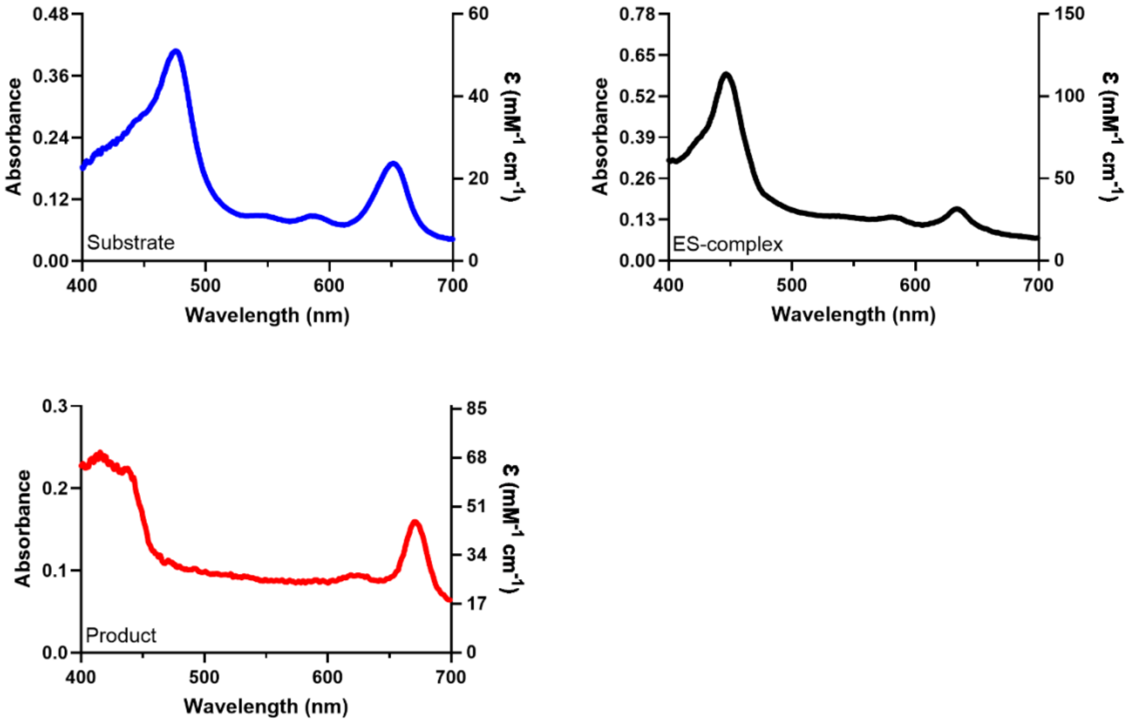

Figure S2. Individual visible spectra for the three species used as reference spectra: Substrate (in blue) , ES-complex (in black) , and Product (in red). All spectra were recorded in buffer with BchNB and BchL present.

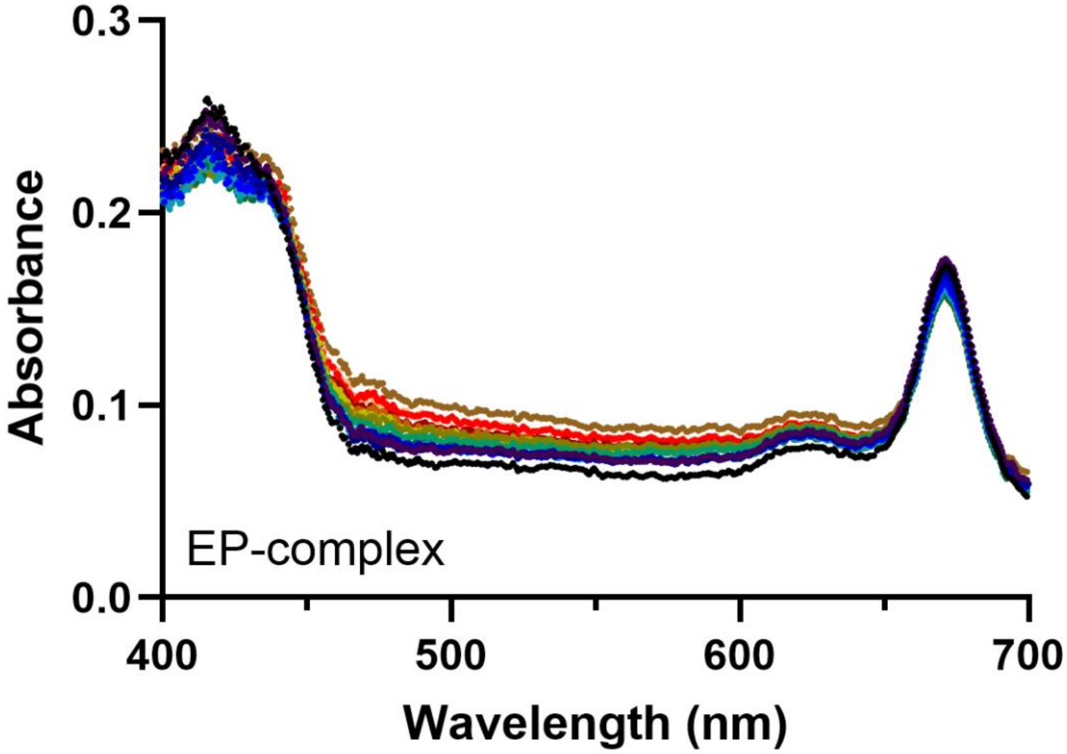

Figure S3. Time-dependent visible spectrum recorded in buffer with BchNB, BchL, and Chlide present.

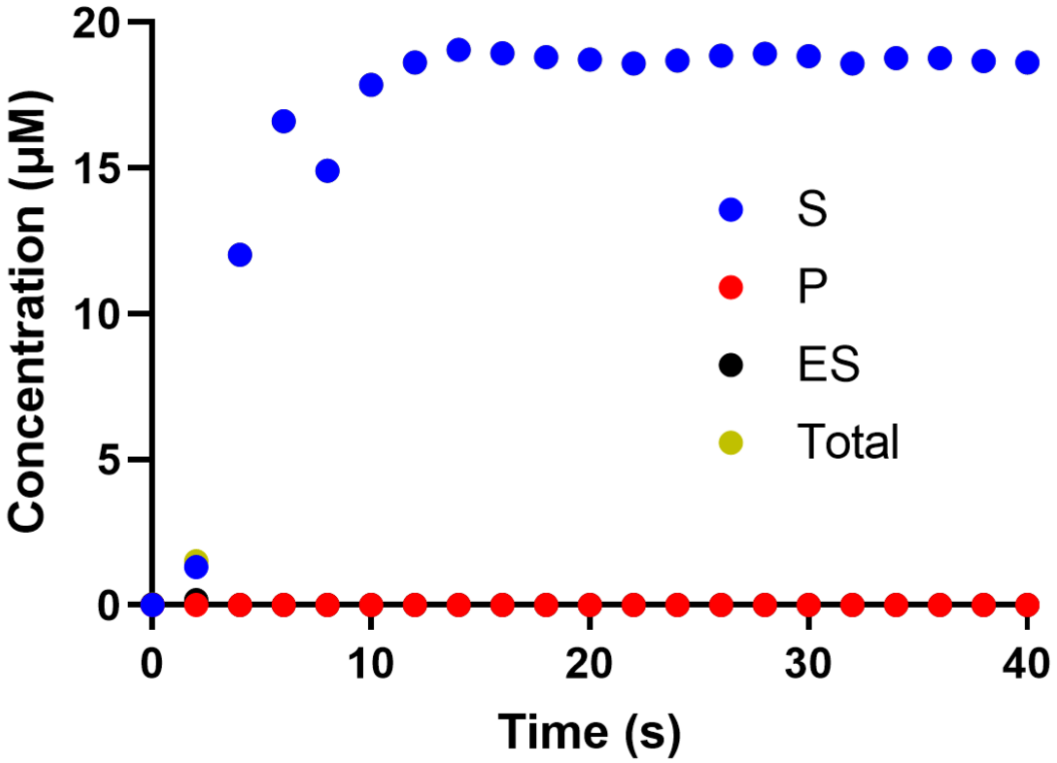

Figure S4. Stabilization of Pchlide in buffer to evaluate the instrument's time response. Measurement of instrument response time following the injection of Pchlide substrate to a stirred solution of buffer (100 mM HEPES buffer (pH 7.5) containing 150 mM NaCl, 3 mM ATP, 10 mM creatine phosphate, 3 mg creatine kinase and 10 mM $MgCl_2$). BchL and BchNB were omitted from this experiment, although the fitting of the ES-complex and P were included as confirmation of the correcting component assignment by our software.
